# Assessing Frailty in the General Medical Clinic of a Tertiary Hospital in Northern Malaysia: The FRAIL Scale or the Clinical Frailty Scale

**DOI:** 10.1155/2021/7570592

**Published:** 2021-08-02

**Authors:** Chiann Ni Thiam, Chin Yik Ooi, Yin Kar Seah, Deik Roy Chuan, Irene Looi, Alan Swee Hock Ch'ng

**Affiliations:** ^1^Department of Medicine, Seberang Jaya Hospital, Perai, Pulau Pinang, Malaysia; ^2^Clinical Research Centre, Seberang Jaya Hospital, Perai, Pulau Pinang, Malaysia

## Abstract

**Background:**

Frailty potentially influences clinicians' decision making on treatment provided they can select the appropriate assessment tools. This study aims to investigate the difference between the FRAIL scale and the Clinical Frailty Scale (CFS) in assessing frailty among community-dwelling older adults attending the General Medical Clinic (GMC) in Seberang Jaya Hospital, Penang, Malaysia.

**Methods:**

The medical records of 95 older patients (age ≥ 65) who attended the GMC from 16 December 2019 to 10 January 2020 were reviewed. Frailty was identified using the FRAIL scale and the CFS. Patient characteristics were investigated for their association with frailty and their difference in the prevalence of frailty by the FRAIL scale and CFS.

**Results:**

The CFS identified nonsignificant higher prevalence of frailty compared to the FRAIL scale (21/95; 22.1% vs. 17/95; 17.9%, ratio of prevalence = 1.235, *p*=0.481). Minimal agreement was found between the FRAIL scale and the CFS (Kappa = 0.272, *p* < 0.001). Three out of 5 components of the FRAIL scale (resistance, ambulation, and loss of weight) were associated with frailty by the CFS. Higher prevalence of frailty was identified by the CFS in those above 70 years of age. The FRAIL scale identified more patients with frailty in ischaemic heart disease patients.

**Conclusion:**

Patient characteristics influenced the choice of the frailty assessment tool. The FRAIL scale and the CFS may complement each other in providing optimized care to older patients who attended the GMC.

## 1. Introduction

Frailty has been an emerging concept that elucidates the health concern of heterogeneous older adults. It is a state of increased vulnerability to adverse outcomes contributed by cumulative declines across multiple physiological systems [1]. It is predictive of various adverse outcomes including procedural complications, falls, institutionalization, disability, and death [2]. Besides that, frailty is dynamic as it transitions between different frailty states over time, and transitions to a greater frailty state were more common [3].

To date, most local studies that targeted the general population gave a prevalence of frailty and prefrailty ranging from 5.7% to 18.3% [4–8] and 61.7% to 72.8%, respectively [6–8]. Recognition of frailty in the general medical clinic facilitates prognostication and patient-centered care implementation for patients with chronic medical illnesses including diabetes, hypertension, and heart failure [9–11]. However, there is paucity of data on frailty in the tertiary medical outpatient clinic in Malaysia.

The growing interest in frailty has led to the budding of frailty assessment tools which may create more confusion for clinicians who intended to incorporate frailty into their practice [12]. Fried Frailty Phenotype, which is the most used approach to describe frailty based on the currently available literature, identifies a person to be frail when he/she meets three out of five phenotypic criteria: low grip strength, low energy, slowed walking speed, low physical activity, and/or unintentional weight loss [1, 13]. However, it is not feasible in an outpatient clinic that is not equipped with a hand-held dynamometer to measure grip strength and clinicians may not be familiar with quantifying physical activity per week. Despite evidence on the validity and predictability, questions arise on how to choose an assessment tool that matches the resources, objectives, and characteristics of the patients in the given clinical setting [2, 14]. This study aimed to investigate the difference between two frailty assessment tools adopting different approaches, namely, the FRAIL scale and the Clinical Frailty Scale (CFS), in assessing frailty among community-dwelling older adults attending the General Medical Clinic (GMC).

## 2. Methods

### 2.1. Study Population and Data Collection

This is a retrospective study on community-dwelling older adults aged 65 and above, who attended the GMC in Seberang Jaya Hospital from 16th December 2019 to 10th January 2020. Seberang Jaya Hospital is a government specialist hospital located in Perai, mainland Penang, Northern Malaysia, serving a local catchment area of 800,000 people. The GMC in Seberang Jaya Hospital which is led by internal medicine specialists runs twice a week.

Medical records and clinical follow-up notes were reviewed. Data collected included age, gender, ethnicity, educational level, comorbidities, number of medications, and blood parameters. The comorbidity burden was expressed as an age-adjusted Charlson Comorbidity Index (CCI) [15]. Polypharmacy is defined as the regular use of at least five medications [16]. Comprehensive Geriatric Assessment (CGA) provided information on the presence of sensory deprivations such as vision or hearing impairment, hospitalisations or falls in the previous year, weight loss, urinary incontinence, self-reported physical fitness and health, social support, and mood. The Lawton Instrumental Activities of Daily Living (IADL) Scale which is usually incorporated into CGA, evaluates patients' ability to perform the following: to use telephone, to shop, to prepare food, to do housekeeping, to do laundry, to travel independently or by public transport, to be responsible for their own medication, and to handle finance [17].

### 2.2. Frailty Assessment Tools

#### 2.2.1. FRAIL Scale

The FRAIL scale is a rapid self-reported screening tool adopting the physical phenotype model [18, 19]. It gave similar predictive accuracy to Fried Frailty Phenotype [18, 19]. Since its introduction in 2008, it gained increased popularity among clinicians in Asian countries [20, 21]. The FRAIL scale consists of 5 components: (1) fatigue (feel tired all or most of the time); (2) resistance (difficult to walk up 10 steps without rest and unaided); (3) ambulation (difficult to walk several hundred yards unaided); (4) illness (more than 4 of the following illnesses: hypertension, diabetes, cancer other than minor skin cancer, chronic lung disease, heart attack, congestive heart failure, angina, asthma, arthritis, stroke, and kidney disease.); and (5) loss of weight (more than 5% loss of weight) [19]. The FRAIL scale gives a score ranges from 0 to 5, 1 point for each component, and it categorizes people to be robust, prefrail, and frail if they score 0, 1 to 2, 3, or above, respectively [19].

#### 2.2.2. Clinical Frailty Scale (CFS)

The Canadian Study of Health and Aging (CSHA) CFS is a 9-point assessment tool that enables clinicians to identify and assess the grade of frailty through routine clinical assessment [22]. A score of 1 to 9 is given according to the description of activities and functional status: 1: very fit (robust people who exercise regularly, and they are among the fittest for their age); 2: well (active people without active disease symptoms but less fit than 1); 3: managing well (people who have well-controlled medical problems but not regularly active); 4: vulnerable (people whose activities limited by symptoms such as being slow or tired, but are still independent); 5: mildly frail (people who have more evident slowing and are dependent on the IADL); 6: moderately frail (people who need help with all outside activities and some personal care); 7: severely frail (people who need help on all personal care but not at high risk of dying within 6 months); 8: very severely frail (people who are completely dependent and approaching the end of life); and 9: terminally ill (people who are not frail evidently but have a life expectancy below 6 months) [22]. The CFS evaluates the physical, medical, and psychosocial aspects of an older adult, but its accuracy relies on the clinician's judgment [22]. The cutoff point for frailty was CFS ≥5 [22].

### 2.3. Statistical Analysis

Analyses were performed using SPSS version 20. Descriptive data were expressed as mean and standard deviation (SD) or median and interquartile range (IQR) for continuous variables, depending on their distribution, and as frequencies and percentages for categorical variables. Associations between categorical variables were assessed using Pearson's chi-squares tests or Fisher's exact test, while the associations between continuous variables were assessed using the independent *T*-tests or Mann–Whitney U test depending on the distribution. Paired categorical variables were assessed using McNemar's test, and the results were expressed as the ratio of prevalence (CFS : FRAIL scale). Cohen's kappa coefficient was used to assess agreement between the FRAIL scale and the CFS. Age was dichotomized into 70 years of age and below and above 70 years of age according to the median of the patients. Analyses were performed to investigate the differences between the FRAIL scale and CFS: the prevalence and associating factors of frailty; patient characteristics; and frailty phenotypes that contribute to inconsistency in the prevalence of frailty. The significance level of all the statistical tests is set at *p* < 0.05. Analyses were only performed on available data.

### 2.4. Ethics Statement

This study was conducted following the ethical principles outlined in the Declaration of Helsinki and Malaysian Good Clinical Practice Guideline. The research protocol was approved by the Medical Research Ethics Committee (MREC), Ministry of Health, Malaysia (government approval number: NMRR-19-3952-52222 IIR).

## 3. Result

In 95 community-dwelling older adults aged 65 and above who attended the GMC, the median age was 71.0 (IQR 67.0–75.0) with similar gender distribution (Table 1). The baseline demographic data, comorbidities, blood parameters, frequency, and rates of geriatric syndromes such as sensory deprivations, polypharmacy, low mood, poor social support, urinary incontinence, falls, and hospital admission are shown in Table 1.

**Figure 1:**
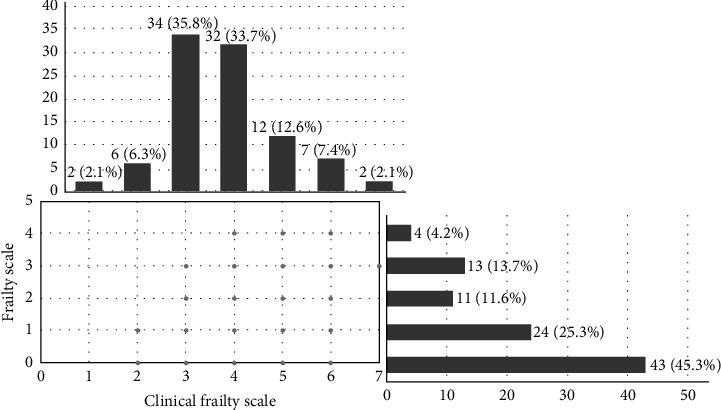
The FRAIL scale and the clinical frailty scale of community-dwelling older adults who attended GMC. FRAIL scale: 0 = robust; 1–2 = prefrail; and ≥3 = frail. Clinical frailty scale: 1 = very fit; 2 = well; 3 = managing well; 4 = vulnerable; 5 = mildly frail; 6 = moderately frail; 7 = severely frail; 8 = very severely frail; and 9 = terminally ill.

We identified 43(45.3%), 35(36.8%), and 17(17.9%) patients to be robust, prefrail, and frail according to the FRAIL scale. The FRAIL scale of the patients is shown in Figure 1. Using the CFS, we found 21(22.1%) patients to be frail and 74(77.9%) to be nonfrail. The CFS of the patients is demonstrated in Figure 1.

### 3.1. Differences between the FRAIL Scale and the Clinical Frailty Scale

#### 3.1.1. Prevalence

CFS identified a nonsignificantly higher prevalence of frailty (22.1% versus 17.9%, ratio of prevalence = 1.235, *p*=0.481) but lesser “vulnerable” patients compared to prefrail patients on the FRAIL scale (33.7% versus 36.8%, ratio of prevalence = 0.916, *p*=0.755). However, there was minimal agreement between the FRAIL scale and the CFS (kappa = 0.272, *p* < 0.001) (Table 2). For instance, two patients who were robust based on the FRAIL scale were found to have a CFS of 5 and 6; one patient who was frail according to the FRAIL scale had a CFS of 3 (Figure 1).

#### 3.1.2. Associated Factors of Frailty

Age, diabetes mellitus, ability to perform IADL, lower haemoglobin level, higher urea level, and lower albumin level were associated with frailty based on both scales (Table 1). Ethnicity, ischaemic heart disease (IHD), chronic kidney disease, congestive cardiac failure, polypharmacy, and hospital admission were the associated factors of frailty by the FRAIL scale only (Table 1). Stroke, visual and hearing impairment, urinary incontinence, and low mood were associated with frailty by the CFS only (Table 1).

#### 3.1.3. Patient Characteristics and Their Prevalence of Frailty

In patients aged above 70 years, the CFS identified significant higher prevalence of frailty (19/48; 39.6% vs 11/48; 22.9%, ratio of prevalence = 1.729, *p*=0.039). Nonsignificantly, more frail patients by the CFS were observed in Malays, patients with history of stroke, visual impairment, and hearing impairment (Table 3). However, the CFS identified a significantly lower prevalence of frailty in IHD patients. (8/48; 16.7% vs. 15/48; 31.3%, ratio of prevalence = 0.534, *p*=0.016) (Table 3). No significant difference in the prevalence of frailty was observed in different gender, educational levels, and comorbidities except those mentioned earlier (Table 3).

### 3.2. Components of the FRAIL Scale

“Resistance” (32/95; 33.7%) was the most scored individual component of the FRAIL scale. The frequencies and rates of other components are shown in Table 4. “Resistance,” “ambulation,” and “loss of weight” were the components of the FRAIL scale that were associated with frailty by the CFS whereas “fatigue” and illness” were not (Table 4).

The differences in the prevalence of frailty between patients who scored “1” and “0” for each component of the FRAIL scale are demonstrated in Table 4.

## 4. Discussion

The CFS identified a nonsignificantly higher rate of frailty compared to the FRAIL scale. However, the CFS demonstrated minimal agreement with the FRAIL scale and there were discrepancies in the prevalence of frailty in certain patient characteristics by different frailty assessment tools.

The finding of minimal agreement between the two scales is consistent with the existing literature as different approaches have been used to conceptualise frailty [23]. The choice of frailty assessment tool may subject older patients to different chronic illness treatment strategies and goals. Our findings which showed that one tool is more sensitive than the other in identifying frailty in patients with specific conditions not only may explain but also guide the approach to deal with these discrepancies. Patients with IHD may appear well on clinician's judgment but they are more likely to report poorer health-related quality of life [24, 25]. This explained why the prevalence of IHD patients living with frailty was higher by the FRAIL scale which is a self-reported tool. Despite inconsistent evidence found on the association between age and self-rated health [25], cognitive impairment and sensory deprivation which are more common with age may have limited the sensitivity of the FRAIL scale in the older group. Therefore, objective evaluation is crucial and preferred over self-rating tool in determining the frailty status among the older group.

Regarding individual components of the FRAIL scale, although “resistance” was the most frequent scored component, overestimation of resistance was still suspected as 6 out of 21 frail patients identified by the CFS did not score for this component. It is questionable that a person with CFS ≥5 can walk 10 steps without rest and unaided. The accuracy of the FRAIL scale relies on the patient's understanding and interpretation of the question. The “illness” component of the FRAIL scale did not reflect well on frailty in this cohort. Besides lack of association, a significantly higher prevalence of frailty by the CFS was found in those who have 4 or fewer comorbidities listed in the FRAIL scale.

We were unable to report the sensitivity and specificity of each tool due to a lack of reference tools, but this is not the objective of our study. Instead, we would like to focus on the practical aspect of frailty assessment, particularly in the tertiary outpatient clinic. Moreover, there is no gold standard assessment of frailty [14]. The time required for each assessment tool was not captured. This can be practically difficult in a medical clinic setting as the clinician tends to explore more history when a deficit is detected. For example, a clinician will enquire on the reason and duration of fatigue instead of moving on to ask the next component of the FRAIL scale.

The main limitations of this study were the small sample size and being a single-site retrospective study. Furthermore, our population was skewed toward a population with comorbidity and, hence, the prevalence reported is not generalisable to other settings. The findings were also limited by the dependency on documentation in clinical notes. The cognitive assessment result was not routinely available, and we could not determine the difference in prevalence between patients with or without cognitive impairment. This study included all the community-dwelling older patients who attended GMC during the study period which meant there was no selection bias. Nevertheless, this is the first study on frailty assessment based in a tertiary outpatient clinic in Malaysia instead of in the community and specifically studied the practical aspect of frailty assessments. The finding of different frailty prevalence, in particular patient characteristics, may help clinicians to gain insight on frailty assessment tool selection in the medical clinic setting and subsequently tailor the management which is both clinically and cost effective according to frailty status. A qualitative study on the opinion and experience with frailty assessment in medical outpatient clinics may have its value for future research.

## 5. Conclusions

Frailty as a modern geriatric giant has attracted clinicians to embrace this concept in decision making. Due to minimal agreement between the two assessment tools, the FRAIL scale and the CFS should not be used interchangeably. Our findings supported that patient characteristic affects the choice of frailty assessment tools in addition to the feasibility and objective in the given clinical setting. We concluded that the FRAIL scale and CFS complement each other to aid the clinician in providing high-quality individualized care for older patients in GMC.

## Figures and Tables

**Table 1:** Characteristics of community-dwelling older adults who attended general medical clinic.

	FRAIL scale			Clinical frailty scale		
	All, *N* = 95	Frail, *N* = 17	Not frail, *N* = 78	*p*	Frail, *N* = 21	Not frail, *N* = 74	*p*
	*n* (%)	*n* (%)	*n* (%)		*n* (%)	*n* (%)	
Age (year)^a^	71.0 (67.0–75.0)	77 (68.5–84.0)	69.5 (67.0–73.0)	0.010	79.0 (72.5–84.0)	69.0 (67.0–72.0)	<0.001
Gender (female)	45 (47.4%)	8 (47.1%)	37 (47.4%)	0.977	10 (47.6%)	35 (47.3%)	0.979

Ethnicity							
Malay	34 (35.8%)	1 (5.9%)	33 (42.3%)	0.006	5 (23.8%)	29 (39.2%)	0.348
Chinese	42 (44.2%)	9 (52.9%)	33 (42.3%)		12 (57.1%)	30 (40.5%)	
Indian	19 (20.0%)	7 (41.1%)	12 (15.4%)		4 (19.0%)	15 (20.3%)	

Educational level							
Never attended school/primary school	52 (54.7%)	12 (70.6%)	30 (38.5%)	0.147	14(66.7%)	38 (51.4%)	0.213
Secondary school/tertiary education	43 (45.3%)	5 (29.4%)	38 (48.7%)		7(33.3%)	36 (48.6%)	

Comorbidities							
Hypertension	80 (84.2%)	17 (100%)	63 (80.8%)	0.065	19 (90.5%)	61 (82.4%)	0.509
Diabetes mellitus	53 (55.8%)	15 (88.2%)	38 (48.7%)	0.003	16 (76.2%)	37 (50.0%)	0.033
Ischaemic heart disease	48 (50.5%)	15 (88.2%)	33 (42.3%)	0.001	8 (38.1%)	40 (54.1%)	0.197
Chronic kidney disease	33 (34.7%)	10 (58.8%)	23 (29.5%)	0.021	10 (47.6%)	23 (31.1%)	0.160
Arthritis	30 (31.6%)	7 (41.2%)	23 (29.5%)	0.347	8 (38.1%)	22 (29.7%)	0.467
Congestive cardiac failure	23 (24.2%)	10 (58.8%)	13 (16.7%)	0.001	8 (38.1%)	15 (20.3%)	0.092
Stroke	19 (20.0%)	5 (29.4%)	14 (17.9%)	0.284	9 (42.9%)	10 (13.5%)	0.003
Charlson Comorbidity Index^b^	5.0 (2.0)	7.2 (2.1)	4.8 (1.6)	<0.001	6.8 (2.2)	4.8 (1.6)	<0.001

IADL^c^							
Lawton IADL Scale^a^	7.0 (5.0–8.0)	4.0 (2.0–7.0)	7.0 (6.0–8.0)	0.001	4.0 (1.0–4.0)	8 (6.8–8.0)	<0.001

Other geriatric syndromes and psychosocial issues							
Visual impairment	32 (33.7%)	8 (47.1%)	24 (30.8%)	0.198	12 (57.1%)	20 (27.0%)	0.010
Hearing impairment	21 (22.1%)	4 (23.5%)	17 (21.8%)	1.000	9 (42.9%)	12 (16.2%)	0.016
Polypharmacy	77 (81.1%)	17 (100.0%)	60 (76.9%)	0.036	19 (90.5%)	58 (78.4%)	0.344
Urinary incontinence	13 (13.7%)	4 (23.5%)	9 (11.5%)	0.24	7 (33.3%)	6 (8.1%)	0.007
Fall ≥1 for the previous year	19 (20.0%)	4 (23.5%)	15 (19.2%)	0.74	7 (33.3%)	12 (16.2%)	0.120
Admission ≥1 for the previous year	21 (22.1%)	8 (47.1%)	13 (16.7%)	0.011	7 (33.3%)	14 (18.9%)	0.231
Low mood	29 (30.5%)	7 (41.2%)	22 (28.2%)	0.293	11 (52.4%)	18 (24.3%)	0.014
Poor social support	5(5.3%)	2 (11.8%)	3 (3.8%)	0.217	1 (4.8%)	4 (5.4%)	1.000

Blood parameters							
Haemoglobin (g/dl)^b^	12.7 (1.8)	11.8 (2.0)	12.9 (1.7)	0.033	11.9 (2.0)	12.9 (1.7)	0.017
Urea (mmol/L)^a^	5.5 (4.3–7.3)	8.0 (5.9–10.3)	5.5 (4.1–6.7)	<0.001	7.2 (4.9–9.4)	5.6 (4.3–6.8)	0.032
Albumin (g/L)^b^	36.1 (4.2)	32.9 (5.3)	37.3 (3.5)	0.005	32.5 (4.7)	37.8 (3.3)	<0.001

^a^Median (IQR), ^b^mean (SD), ^c^instrumental activities of daily living.

**Table 2:** The agreement between the FRAIL scale and clinical frail scale (CFS).

		Clinical frailty scale, *n* (%)			
		1 to 3	4	≥5	Total
FRAIL	Robust	28 (65.1%)	13 (30.2%)	2 (4.7%)	43 (45.3%)
	Prefrail	13 (37.1%)	13 (37.1%)	9 (25.7%)	35 (36.8%)
	Frail	1 (5.8%)	6 (35.3%)	10 (58.8%)	17 (17.9%)
	Total	42 (44.2%)	32 (33.7%)	21 (22.1%)	95
					kappa = 0.272
					(*p* < 0.001)

**Table 3:** Patient characteristics and their prevalence of frailty by the FRAIL scale and the clinical frailty scale.

	*N*	Prevalence of frailty		Ratio of prevalence	*p*
		FRAIL scale, *n* (%)	Clinical frailty scale, *n* (%)		
Age					
≤70	47	6 (12.8%)	2 (4.3%)	0.102	0.219
>70	48	11 (22.9%)	19 (39.6%)	1.729	0.039

Gender					
Female	45	8 (17.8%)	10 (22.2%)	1.247	0.754
Male	50	9 (18.0%)	11 (22.0%)	1.222	0.727

Ethnicity					
Malay	34	1 (2.9%)	5 (14.7%)	5.069	0.125
Chinese	42	9 (21.4%)	12 (28.6%)	1.336	0.508
Indian	19	7 (36.8%)	4 (21.1%)	0.573	0.375

Educational level					
Never attended school/primary school	52	12 (23.1%)	14 (26.9%)	1.165	0.774
Secondary school/tertiary education	43	5 (11.6%)	7 (16.3%)	1.405	0.687

Comorbidities					
Hypertension	80	17 (21.3%)	19 (23.8%)	1.117	0.804
Diabetes mellitus	53	15 (28.3%)	16 (30.2%)	1.067	>0.999
Ischaemic heart disease	48	15 (31.3%)	8 (16.7%)	0.534	0.016
Chronic kidney disease	33	10 (30.3%)	9 (27.3%)	0.901	>0.999
Arthritis	30	7 (23.3%)	10 (26.7%)	1.145	>0.999
Congestive cardiac failure	23	10 (43.5%)	8 (34.8%)	0.800	0.687
Stroke	19	5 (26.3%)	9 (47.4%)	1.802	0.125

Other geriatric syndromes and psychosocial issues					
IADL^a^ dependent	55	14 (25.5%)	20 (24.4%)	0.957	0.180
Visual impairment	32	8 (25.0%)	12 (37.5%)	1.500	0.334
Hearing impairment	21	4 (19.0%)	9 (42.9%)	2.258	0.125
Polypharmacy	77	17 (22.1%)	18 (23.4%)	1.059	>0.999
Urinary incontinence	13	4 (30.8%)	6 (46.2%)	1.500	0.625
Fall ≥1 for the previous year	19	4 (21.1%)	6 (31.6%)	1.497	0.625
Admission ≥1 for the previous year	21	8 (38.1%)	7 (33.3%)	0.874	>0.999
Low mood	29	7 (24.1%)	11 (37.9%)	1.573	0.388
Poor social support	5	2 (40.0%)	1 (20.0%)	0.500	>0.999

^a^Instrumental activities of daily living.

**Table 4:** Components of the FRAIL scale and their association with frailty and prevalence of frailty between patients who scored “1” and “0” for each component of the FRAIL scale.

	FRAIL scale			Clinical frailty scale (CFS)			Prevalence of frailty			
	Frail, *N* = 17	Not frail, *N* = 78	*p*	Frail, *N* = 21	Not frail, *N* = 74	*p*	FRAIL	CFS	Ratio of prevalence	*p*
1. Fatigue										
Score = 1, 18(18.9%)	7 (41.2%)	11 (14.1%)	0.017	5 (23.8%)	13 (17.6%)	0.536	7 (38.9%)	5 (27.8%)	0.715	0.687
Score = 0, 77(81.1%)	10 (58.8%)	67 (85.9%)		16 (76.2%)	61 (82.4%)		10 (13.0%)	16 (20.8%)	1.600	0.146

2. Resistance										
Score = 1, 32(33.7%)	17 (100%)	15 (19.2%)	<0.001	15 (71.4%)	17 (23.0%)	<0.001	17 (53.1%)	15 (46.9%)	0.883	0.774
Score = 0, 63(66.3%)	0	63 (80.8%)		6 (28.6%)	57 (77.0%)		0	6 (9.5%)	N/A^a^	N/A^a^

3. Ambulation										
Score = 1, 19(20.0%)	14 (82.4%)	5 (6.4%)	<0.001	14 (66.7%)	5 (6.8%)	<0.001	14 (73.7)	14 (73.7%)	1.000	>0.999
Score = 0, 76(80.0%)	3 (17.6%)	73 (93.6%)		7 (33.3%)	69 (93.2%)		3 (3.9%)	7 (9.2%)	2.359	0.344

4. Illness										
Score = 1, 19(20.0%)	11 (64.7%)	8 (10.3%)	<0.001	7 (33.3%)	12 (16.2%)	0.120	11 (57.9%)	7 (36.8%)	0.635	0.219
Score = 0, 76(80.0%)	6 (35.3%)	70 (89.7%)		14 (66.7%)	62 (83.8%)		6 (7.9%)	14 (18.4%)	2.329	0.039

5. Loss of weight										
Score = 1, 13(13.7%)	6 (35.3%)	7 (9.0%)	0.011	6 (28.6%)	7 (9.5%)	0.035	6 (46.2%)	6 (46.2%)	1.000	>0.999
Score = 0, 82(86.3%)	11 (64.7%)	71 (91.0%)		15 (71.4%)	67 (90.5%)		11 (13.4%)	15 (18.3%)	1.366	0.424

^a^Not applicable. 1. Fatigue: feel tired all or most of the time; 2. Resistance: difficult to walk 10 steps without rest and unaided; 3. Ambulation: difficult to walk several hundred yards unaided; 4. Illness: more than 4 of the following illnesses: hypertension, diabetes, cancer other than minor skin cancer, chronic lung disease, heart attack, congestive heart failure, angina, asthma, arthritis, stroke, and kidney disease; and 5. Loss of weight: more than 5% loss of weight.

## Data Availability

Data are available only on request, due to privacy concerns and ethical restrictions.
